# Hepatic arterial infusion chemotherapy combined with lenvatinib and PD-1 inhibitors versus lenvatinib and PD-1 inhibitors for unresectable HCC: a meta-analysis

**DOI:** 10.3389/fonc.2024.1500496

**Published:** 2024-12-24

**Authors:** Min Wei, Pengwei Zhang, Chaofeng Yang, Yang Li

**Affiliations:** Sichuan Key Laboratory of Medical Imaging, Department of Radiology, The Affiliated Hospital of North Sichuan Medical College, Nanchong, China

**Keywords:** meta-analysis, hepatocellular carcinoma, HAIC, lenvatinib, PD-1 inhibitors

## Abstract

**Objectives:**

This study aimed to evaluate the effectiveness of combining hepatic arterial infusion chemotherapy (HAIC) with lenvatinib and programmed cell death protein 1 (PD-1) inhibitors in the treatment of advanced, unresectable hepatocellular carcinoma (HCC).

**Methods:**

A comprehensive search across multiple databases was conducted to identify relevant studies published up to May 2024. This search focused on clinical trials investigating the combination of HAIC with lenvatinib and PD-1 inhibitors for the treatment of advanced HCC. Data from these trials were analyzed using either fixed-effects or random-effects models, with results reported as hazard ratios (HRs) or risk ratios (RRs) with 95% confidence intervals (CIs). To evaluate the robustness of the findings, trial sequential analysis was employed.

**Results:**

A total of 8 cohort studies encompassing 1073 patients with unresectable HCC were included. Compared with other treatment regimens, the combined use of HAIC, lenvatinib, and PD-1 inhibitors significantly improved overall survival (OS) (HR=0.53 [95% CI 0.45, 0.63], P<0.00001), progression-free survival (PFS) (HR 0.56 [95% CI 0.46, 0.61], P<0.0001), the objective response rate (ORR) (RR=1.82 [95% CI 1.52, 2.18], P<0.00001), and the disease control rate (DCR) (RR=1.24 [95% CI 1.16, 1.33], P<0.00001). Trial sequential analysis (TSA) results indicated that the existing data were sufficient for making quantitative conclusions about the ORR and DCR.

**Conclusion:**

Combining HAIC with lenvatinib and PD-1 inhibitors enhances the effectiveness of treatment for unresectable HCC. This approach is particularly beneficial for patients who have a high tumor burden or those who are refractory to transarterial chemoembolization (TACE), providing a more effective solution for these challenging cases.

**Systematic review registration:**

https://www.crd.york.ac.uk/prospero/display_record.php?ID=CRD42024575853, identifier CRD42024575853.

## 1 Introduction

Hepatocellular carcinoma (HCC) is a prevalent malignant neoplasm that ranks sixth globally in terms of cancer incidence and ranks third in terms of mortality rate (1). Currently, surgical resection is considered the most effective treatment for hepatocellular carcinoma. Unfortunately, many patients are only diagnosed when the tumor has progressed to an advanced stage, which often makes surgical intervention impractical or unfeasible. For unresectable HCC patients, arterial therapies play a crucial role. These methods primarily include transarterial embolization (TAE), transarterial chemoembolization (TACE), drug-eluting bead transarterial chemoembolization (DEB-TACE), selective internal radiation therapy (SIRT), and hepatic arterial infusion chemotherapy (HAIC) (2). Despite advancements, the prognosis for patients with advanced HCC remains unclear. This situation highlights the critical need for safe and therapeutic systemic therapies to serve as supplementary treatments, addressing the critical gaps in current management options (3). Over the past decade, significant advancements have been made in both systemic and local therapies for HCC. According to the latest guidelines, advanced HCC should be managed with a comprehensive approach that combines local and systemic treatments (4).

Lenvatinib is an oral multikinase inhibitor approved in various countries for first-line treatment of unresectable HCC. Research has shown that lenvatinib can prolong progression-free survival (PFS) and overall survival (OS) in these patients while also increasing objective response rates (ORRs) (5, 6). HAIC delivers chemotherapy drugs directly to the target tumor, increasing local drug concentrations while reducing systemic adverse effects. Additionally, literature reports indicate that lenvatinib can normalize aberrant angiogenesis induced by interventional therapies. Several studies have demonstrated that combining HAIC with targeted therapies yields superior efficacy and safety outcomes (7, 8).

The progression of tumors involves evasion of immune surveillance. For example, programmed cell death protein 1 (PD-1) can inhibit the activation and function of T cells, whereas PD-1 inhibitors can block this evasion mechanism, thereby increasing the immune system’s capacity to target and destroy tumors. In one study, approximately 15% of patients continued to exhibit objective tumor responses (9). However, approximately one-third of HCC patients at this stage are resistant to PD-1 or PD-L1 inhibitors, and in some cases, treatment may even accelerate tumor growth (10). Furthermore, the antitumor effects of these inhibitors as monotherapies are often unsatisfactory (11). But a study by Ren et al. on the combination of immune checkpoint inhibitors (ICIs) and tyrosine kinase inhibitors (TKIs) found positive results (12). Based on these findings, theoretically, combination treatment with hepatic arterial infusion chemotherapy, lenvatinib, and PD-1 inhibitors could achieve more pronounced therapeutic effects in patients with unresectable HCC. However, the evidence supporting this theory remains limited. Therefore, this meta-analysis aims to consolidate the current evidence regarding the efficacy of combining HAIC, lenvatinib, and PD-1 inhibitors for the treatment of unresectable hepatocellular carcinoma. Additionally, this meta-analysis aims to examine whether the gathered information offers a robust foundation for assessing the effectiveness of this combined treatment approach.

## 2 Study design

### 2.1 Search strategy

Extensive article searches were performed across the Embase, PubMed, Web of Science, and Cochrane Central Register of Controlled Trials (Central) databases, encompassing studies published from inception to May 2024. The search strategy involved combinations of keywords related to “hepatic arterial infusion chemotherapy,” “hepatocellular carcinoma,” “immune checkpoint inhibitors,” “PD-1 inhibitors,” and “lenvatinib.” The Embase database utilized the Emtree life sciences thesaurus, whereas the other databases employed Medical Subject Headings (MeSH). Manual retrieval of references and related reviews was performed to identify potentially relevant studies. This meta-analysis was registered with PROSPERO (CRD42024575853).

### 2.2 Study screening

The inclusion criteria were as follows (1): study population: patients diagnosed with HCC confirmed by imaging or pathology; (2) intervention: the experimental groups were treated with a combination of HAIC, lenvatinib, and PD-1 inhibitors; and (3) outcome measures: endpoints in this meta-analysis included overall survival (OS), progression-free survival (PFS), objective response rate (ORR) and disease control rate (DCR), with postintervention OS and PFS evaluated via hazard ratios (HRs); (4) study types: case−control studies, cohort studies, or randomized controlled trials (RCTs). The tumor response was evaluated according to Modified response evaluation criteria in solid tumors (mRECIST) or Response Evaluation Criteria in Solid Tumors (RECIST) version 1.1 (13, 14), and the tumor response was divided into a complete response (CR), partial response (PR), stable disease (SD) and progressive disease (PD). The ORR was defined as the percentage of patients who achieved CR or PR among all patients, and the DCR was defined as the percentage of patients who achieved CR, PR or SD. PFS was defined as the time from the initiation of treatment to the occurrence of disease progression. OS was defined as the time from treatment initiation to cancer-related death.

The exclusion criteria were as follows: (1) case reports, case series, editorials, commentaries, and reviews; (2) irrelevant study content; (3) lack of relevant data and inaccessible; and (4) non-English literature.

### 2.3 Data extraction and evaluation of literature quality

Two researchers individually screened the literature, extracted the data, and cross-verified the data. EndNote X9.3.3 software was used for reference management. In cases of disagreement, resolution involved consulting a third experienced researcher. Data extraction included author names, study type, publication year, patient characteristics (age, sex, and tumor stage), details of the intervention, and outcomes related to tumor control measures.

The quality of the observational studies was evaluated on the basis of the modified Newcastle−Ottawa Scale (NOS), with studies scoring 5 or higher deemed to be of high quality (15).

### 2.4 Statistical analysis

Statistical analysis was performed via Review Manager 5.3. The primary outcomes assessed were OS and PFS, with the results presented as log hazard ratios (log HRs) and standard errors. Heterogeneity among the included studies was evaluated via the Q test and analyzed with I² statistics, where I² values of 25%, 50%, and 75% corresponded to low, moderate, and high levels of heterogeneity, respectively (16). Meta-analyses were performed using a fixed-effects model when P > 0.1 and I^2^ < 50%; otherwise, a random-effects model was used (17, 18). Substantial heterogeneity was investigated through sensitivity analyses involving stepwise exclusion of individual studies. Subgroup analyses were implemented according to the therapy regimens used in the control group. Publication bias was assessed via funnel plots. A two-sided P value < 0.05 was considered statistically significant.

### 2.5 Trial sequential analysis

TSA is a method used to assess the cumulative effect of data in systematic reviews or meta-analyses. Its primary purpose is to avoid increasing the risk of type I errors (false-positives) because of repeated hypothesis testing, particularly when there are limited cumulative data or frequent interim analyses. TSA combines principles from traditional meta-analysis and sequential analysis by defining a predetermined sample information size to evaluate whether the accumulated data are sufficient to draw reliable conclusions. This study utilized TSA software for TSA. After accounting for heterogeneity among the included studies, TSA was set with an overall risk of type I error of 5% and a power of 80%, which represents the optimal sample size estimation for statistical inference in the meta-analysis. The anticipated impact of the intervention was projected by analyzing the effect sizes reported in the studies included in the analysis.

## 3 Results

### 3.1 Study screening

**Figure 1:**
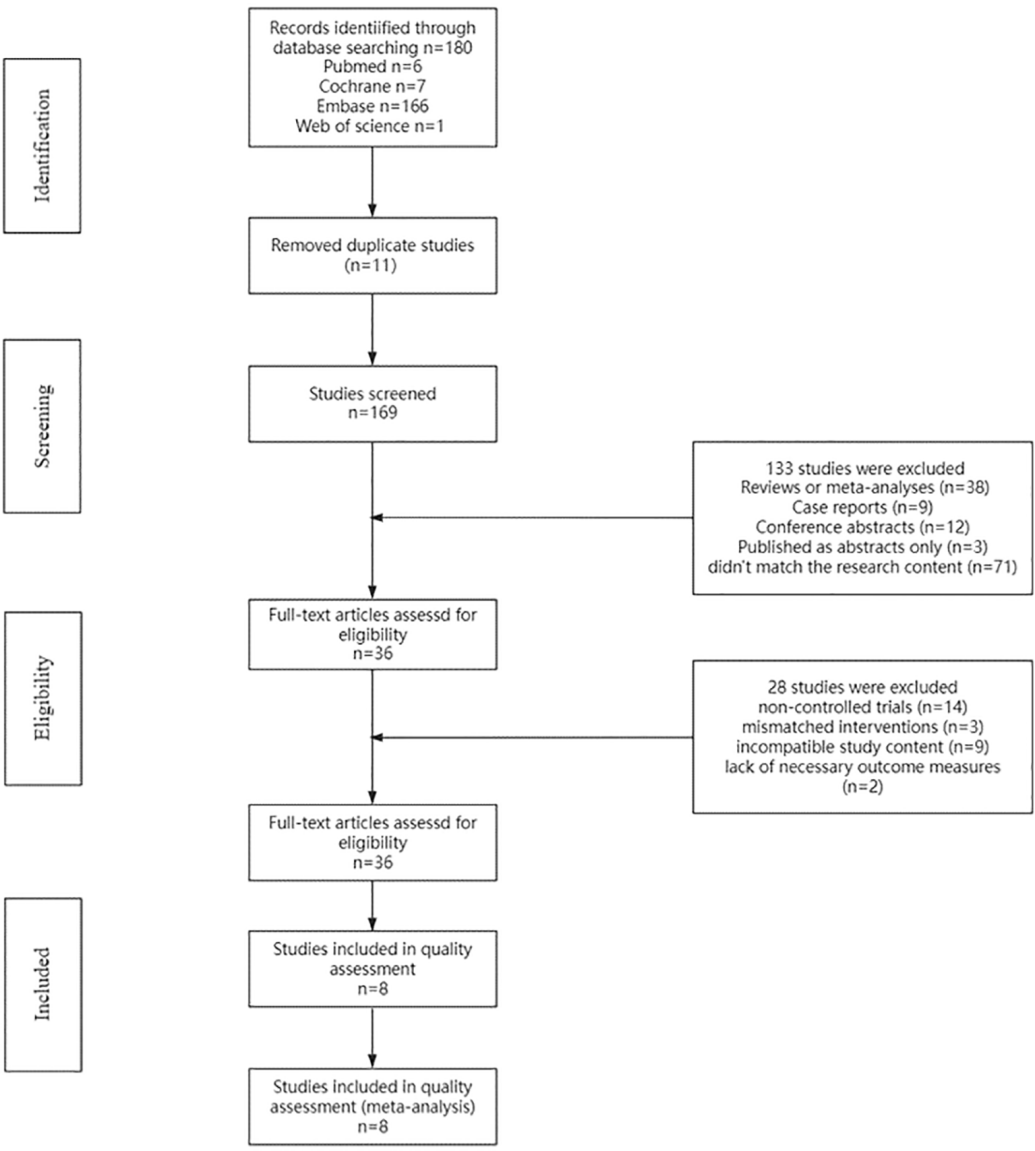
Flow chart.

The PubMed, Embase, Cochrane Central Register of Controlled Trials (Central), and Web of Science databases were searched. Initially, 180 potentially relevant studies meeting the inclusion criteria were identified. After 11 duplicate studies were removed, 169 titles and abstracts were screened. After the titles and abstracts were reviewed, 133 studies were excluded: case reports (n=9), reviews or meta-analyses (n=38), conference abstracts (n=12), studies published as abstracts only (n=3), or studies that did not match the research content (n=71). Finally, 36 studies remained for full-text assessment of eligibility. Following a comprehensive evaluation of the full-text articles, 28 studies were excluded for the following reasons: noncontrolled trials (n=14), mismatched interventions (n=3), incompatible study content (n=9), and lack of necessary outcome measures (n=2). A total of 8 articles (19–26) were ultimately included in the systematic review and meta-analysis (**Figure 1**).

### 3.2 Study characteristics

This meta-analysis summarized 8 studies published from 2021 to 2024, including a total of 1073 patients with unresectable HCC. The detailed study characteristics are presented in **Table 1**. All included studies were retrospective cohort studies, with the majority of patients aged between 50 and 65 years and predominantly male, consistent with the distribution characteristics of hepatocellular carcinoma. These studies utilized various PD-1 inhibitors, including pembrolizumab, nivolumab, sintilimab, toripalimab, camrelizumab, tislelizumab, and sindilizumab. The intervention of interest was the combination of HAIC with lenvatinib and PD-1 inhibitors, whereas control interventions, including HAIC with lenvatinib alone, lenvatinib plus PD-1 inhibitors, lenvatinib alone, HAIC combined with TACE plus PD-1 inhibitors and lenvatinib, and HAIC plus PD-1 inhibitors, varied. The 8 included retrospective cohort studies were assessed via the NOS (**Table 2**), and all were rated as high-quality studies.

**Table 1:** Baseline characteristics of patients in the trials included in the meta-analysis.

Name	Year	Study type	Treatment regimen	PD-1	Study period	No. Patients	Male/female	Age	ECOG	BCLC	NOS score
Lin LW (21)	2023	Retrospective	HAIC+LEN+P/HAIC+LEN	Camrelizumab/Sintilimab	June 2017-July 2022	75	66/9	55.3 ± 9.5	nr	B/C:44/31	7
						74	60/14	56.0 ± 10.5		B/C:41/33	
He MK	2023	Retrospective	HAIC+LEN+P/LEN	Toripalimab	February 2019 to August 2019	71	59/12	≤50/>50:40/31	0/1:14/57	nr	8
						86	77/9	≤50/>50:42/44	0/1:22/46		
Diao LF (22)	2024	Retrospective	HAIC+LEN+P/LEN+P	Sintilimab,/Camrelizumab/Pembrolizumab/Tislelizumab	2020 to 2022	58	49/9	≤50/>50:16/42	0-1/2:27/31	B/C:24/34	6
						63	50/13	≤50/>50:14/49	0-1/2:23/40	B/C:25/38	
Mei J (19)	2021	Retrospective	HAIC+LEN+P/LEN+P	nr	July 2018 to December 2019	45	38/7	49.1 ± 10.6	nr	B/C:5/40	7
						25	18/7	50.1 ± 12.3		B/C:7/18	
Chen S (23)	2023	Retrospective	HAIC+LEN+P/TACE+HAIC+LEN+P	Tislelizumab	January 2019 to February 2022	50	46/4	55 (36–71)	nr	A/B:35/15	6
						50	42/8	56 (43–62)		A/B:36/14	
Guan RG (25)	2024	Retrospective	HAIC+LEN+P/LEN+P	Pembrolizumab/Nivolumab/Sintilimab/Toripalimab/Camrelizumab	January 2019 to December 2022	127	107/20	51.9 ± 10.9	nr	nr	7
						103	94/9	54.0 ± 11.5			
Chen S (23)	2021	Retrospective	HAIC+LEN+P/LEN+P	Pembrolizumab	March 2018 to March 2021	84	72/12	52 (42–67)	0/1:38/46	B/C:22/62	6
						86	71/15	53 (43–69)	0/1:35/51	B/C:21/65	
Yu WC (26)	2023	Retrospective	HAIC+LEN+P/HAIC+P	Sindilizumab/Camrelizumab/Tislelizumab/Pembrolizumab	January 2019 to March 2022	39	37/2	50.9 ± 10.9	0-1/2:36/3	nr	8
						37	35/2	47.9 ± 11.0	0-1/2:34/3		

HAIC, hepatic arterial infusion chemotherapy; LEN, lenvatinib; P, PD-1 inhibitors; TACE, trans-arterial chemoembolization; ECOG, Eastern Oncology Group; BCLC, Barcelona Clinic Liver cancer; NOS, Newcastle-Ottawa Quality Assessment Scale.

**Table 2:** NOS quality assessment results of studies.

Name	Year	Selection	Comparability	Outcome	Total
Lin LW (21)	2023	★★★	★	★★★	7
He MK	2023	★★★★	★	★★★	8
Diao LF (22)	2024	★★★	★	★★	6
Mei J (19)	2021	★★★	★	★★★	7
Chen S (23)	2023	★★★	★	★★	6
Guan RG (25)	2024	★★★	★	★★★	7
Chen S (24)	2021	★★★	★	★★	6
Yu WC (26)	2023	★★★★	★	★★★	8

### 3.3 Progression-free survival

**Figure 2:**
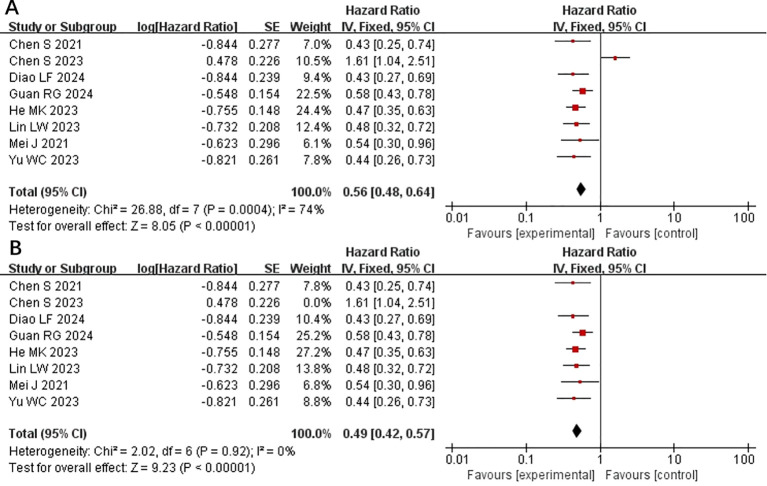
Forest plot of meta-analysis of progression-free survival (PFS). **(A)** Forest plot of meta-analysis of PFS of all included studies. **(B)** Forest plot of meta-analysis of PFS after excluding Chen et al. (2023).

All included studies provided PFS data (15–22). Heterogeneity testing (I^2^ = 74% <50%, P=0.0004 <0.1) revealed significant heterogeneity among the selected studies, warranting further investigation into the sources of heterogeneity (**Figure 2A**). A sensitivity analysis of the 8 studies revealed that the study by Chen et al. (19) significantly influenced heterogeneity. After excluding this study and reassessing heterogeneity, the results revealed no significant heterogeneity among the remaining 7 studies (I^2^ = 0% <50%, P=0.92 >0.1). Using a fixed-effects model to combine the effect sizes of these 7 studies, the results indicated that HAIC combined with lenvatinib and PD-1 inhibitors could prolong PFS (HR 0.56 [95% CI 0.46, 0.61], P < 0.0001) (**Figure 2B**). In subsequent meta-analysis, we excluded the study by Chen et al. (19) due to its significant impact on heterogeneity, as it presented results in a direction contrary to those of other studies.

**Figure 3:**
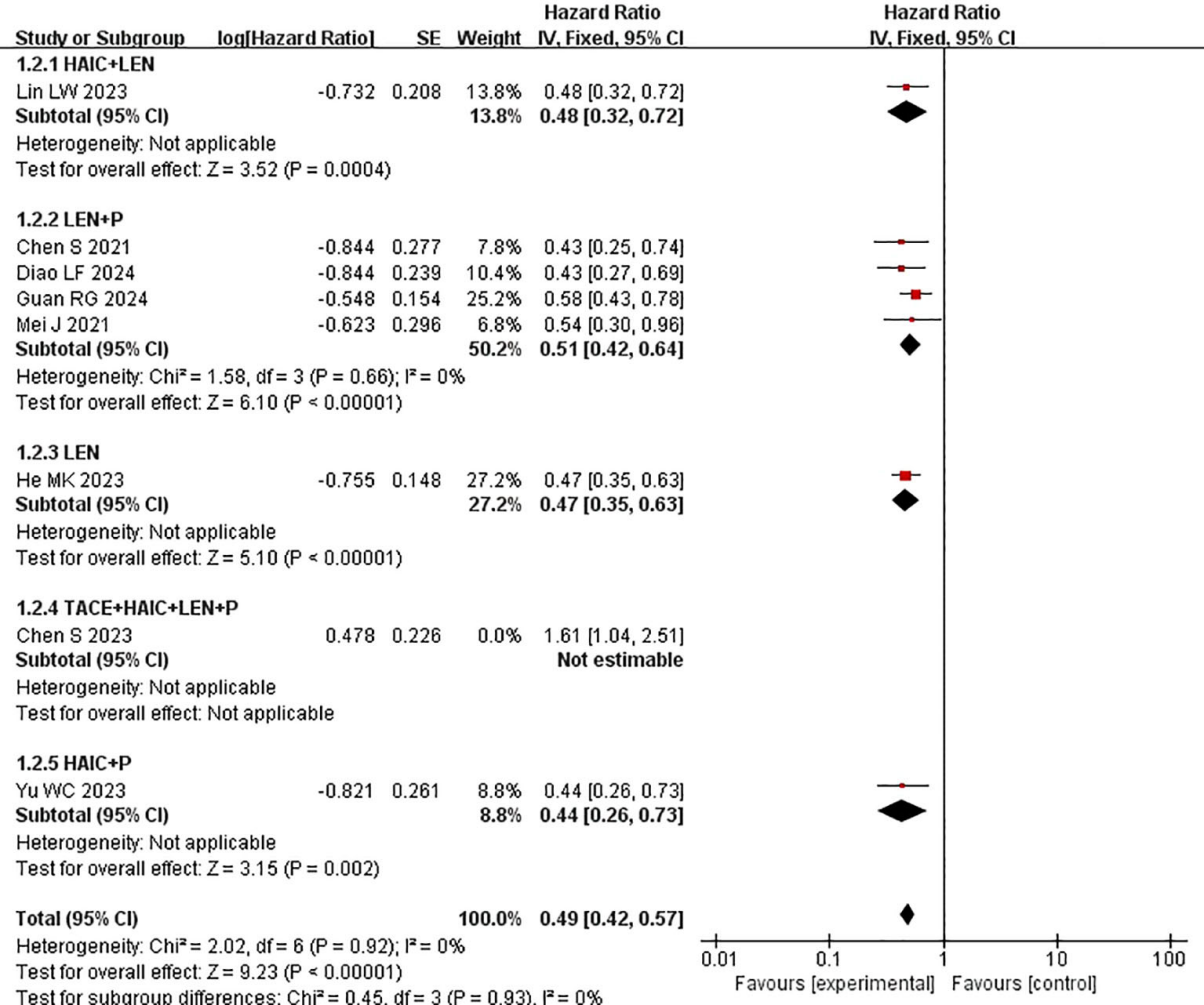
Forest plot of the meta-analysis of progression-free survival in subgroups (PFS).

Subgroup analysis of the control group interventions revealed that, compared with HAIC + lenvatinib (HR=0.46 [95% CI 0.30, 0.71]; P=0.004), lenvatinib + PD-1 inhibitors (HR=0.44 [95% CI 0.30, 0.65]; P<0.0001), lenvatinib monotherapy (HR=0.47 [95% CI 0.35, 0.63]; P<0.00001), and HAIC + PD-1 inhibitors (HR=0.44 [95% CI 0.26, 0.73]), the combination of HAIC with lenvatinib and PD-1 inhibitors significantly improved PFS (**Figure 3**).

### 3.4 Overall survival

**Figure 4:**
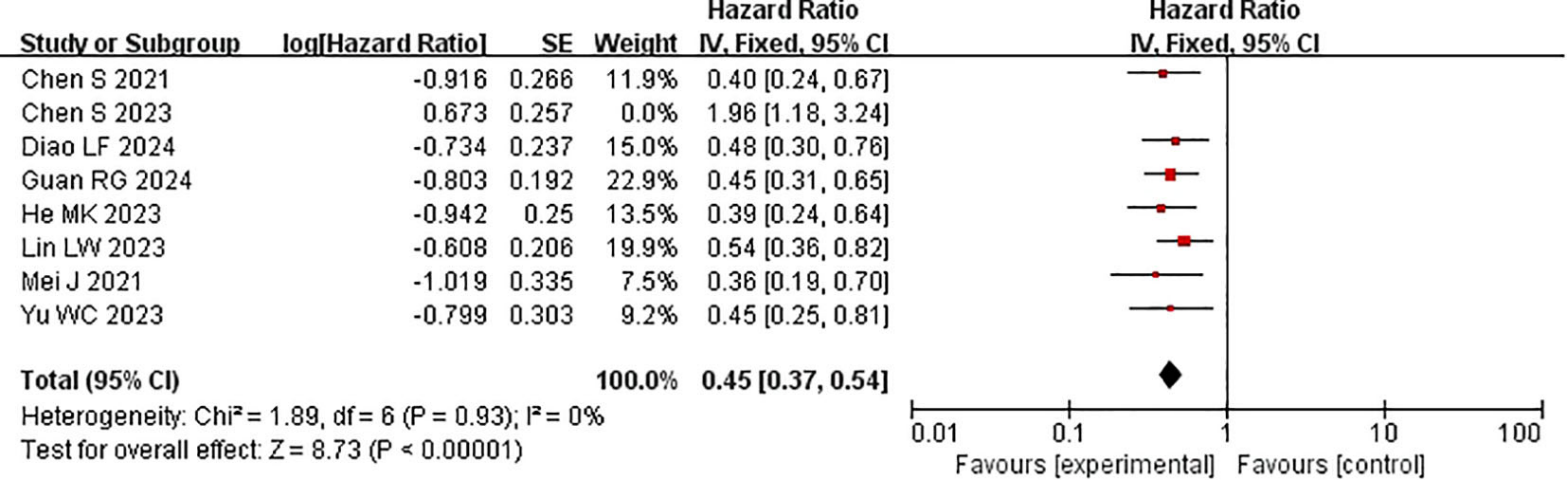
Forest plot of meta-analysis of overall survival (OS).

Every study included in the analysis provided data on OS (15–22). Heterogeneity testing (I^2^ = 77%, P<0.0001) revealed significant heterogeneity among the selected studies, warranting further investigation into the sources of heterogeneity. The sensitivity analysis of the 8 studies indicated that the study by Chen et al. (19) significantly influenced heterogeneity. After excluding this study and reevaluating heterogeneity, the results revealed no significant heterogeneity among the remaining 7 studies (I^2^ = 0% <50%, P=0.9>0.1). A fixed-effects model was used to combine the effect sizes of these 7 studies. The results demonstrated that the combination of HAIC with lenvatinib and PD-1 inhibitors significantly improved OS (HR=0.53 [95% CI 0.45, 0.63], P<0.00001) compared with other treatments (**Figure 4**).

**Figure 5:**
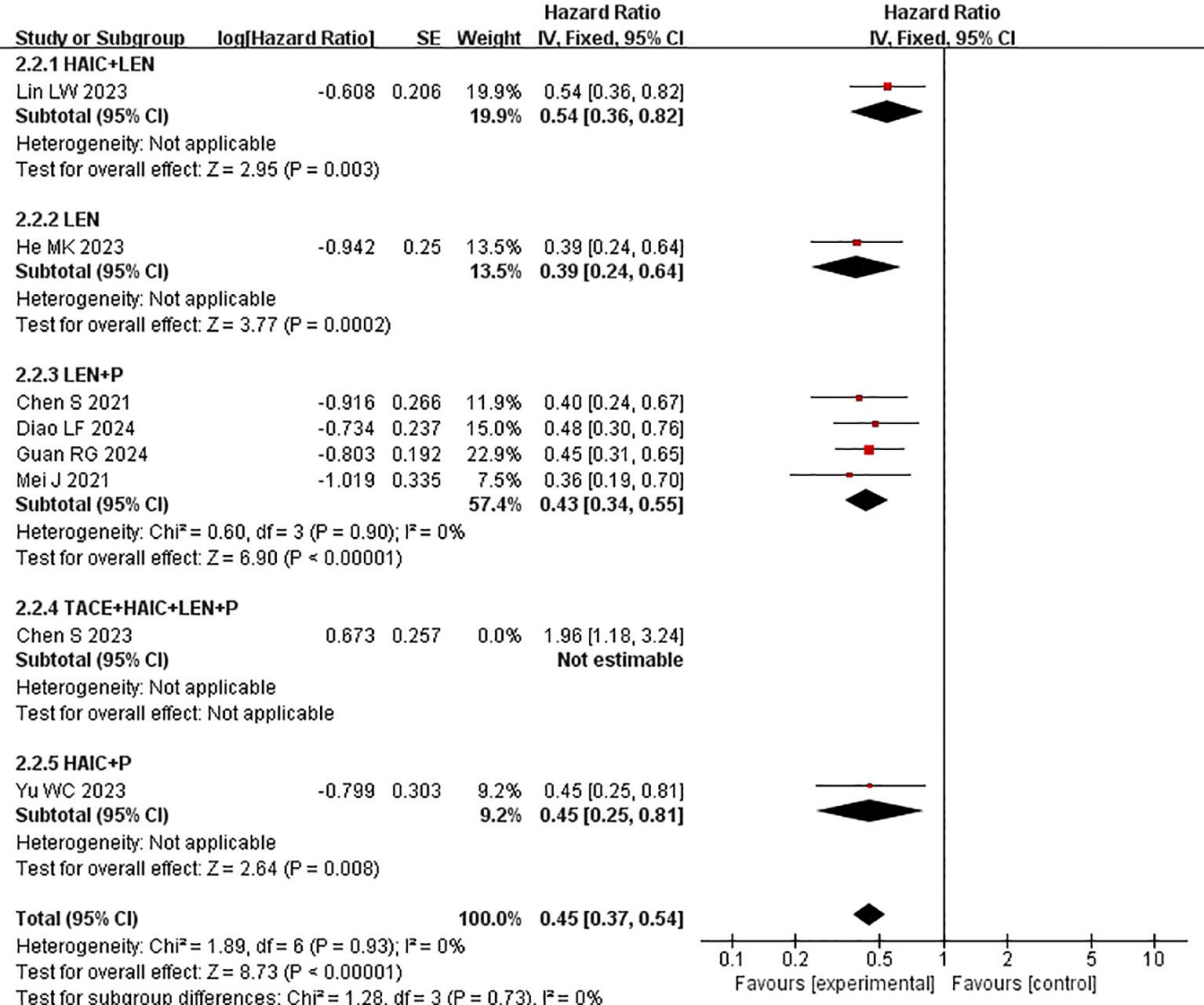
Forest plot of the meta-analysis of overall survival in subgroups.

Subgroup analysis of the control group interventions revealed that, compared with HAIC + lenvatinib (HR=0.54 [95% CI 0.49, 0.61]; P<0.00001), lenvatinib + PD-1 inhibitors (HR=0.42 [95% CI 0.35, 0.50]; P<0.0001), lenvatinib monotherapy (HR=0.39 [95% CI 0.33, 0.46]; P<0.00001), and HAIC + PD-1 inhibitors (HR=0.45 [95% CI 0.33, 0.61]), the combination of HAIC with lenvatinib and PD-1 inhibitors significantly improved PFS (**Figure 5**).

### 3.5 Objective response rate

**Figure 6:**
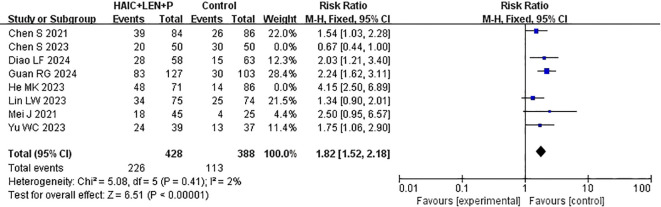
Forest plot of meta-analysis of objective response rate (ORR).

ORR data were reported in every study that was included in the analysis (15–22). After heterogeneity testing (I^2^ = 82%, P<0.00001), significant heterogeneity was detected among the selected studies, warranting further investigation into the sources of heterogeneity. The sensitivity analysis of the 8 studies indicated that the study by Chen et al. (19) and He et al. (16) significantly influenced heterogeneity. After excluding these study and reevaluating heterogeneity, the results revealed no significant heterogeneity among the remaining 6 studies (I^2^ = 2% <50%, P =0.41>0.1). A fixed-effects model was used to combine the effect sizes of these 6 studies, and the results demonstrated that HAIC combined with lenvatinib and PD-1 inhibitors achieved a better ORR (RR=1.82 [95% CI 1.52, 2.18], P<0.00001) (**Figure 6**).

### 3.6 Disease control rate

**Figure 7:**
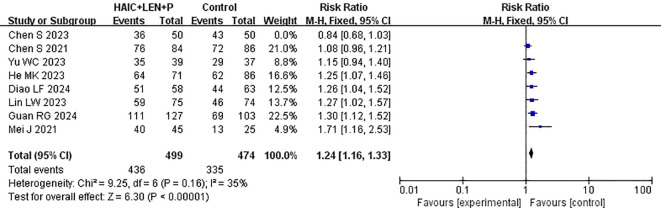
Forest plot of meta-analysis of disease control rate (DCR).

Data on DCR were provided by each of the studies included in the analysis (15–22). Heterogeneity testing (I^2^ = 65%, P=0.005) revealed significant heterogeneity among the selected studies, warranting further investigation into the sources of heterogeneity. The sensitivity analysis of the 8 studies indicated that Chen et al. (19) significantly influenced heterogeneity. After excluding this study and reevaluating heterogeneity, the results revealed no significant heterogeneity among the remaining 7 studies (I^2^ = 35% <50%, P =0.16>0.1). A fixed-effects model was used to combine the effect sizes of these 7 studies, and the results demonstrated that HAIC combined with lenvatinib and PD-1 inhibitors achieved a better DCR (RR=1.24 [95% CI 1.16, 1.33], P<0.00001) (**Figure 7**).

### 3.7 Trial sequential analysis

**Figure 8:**
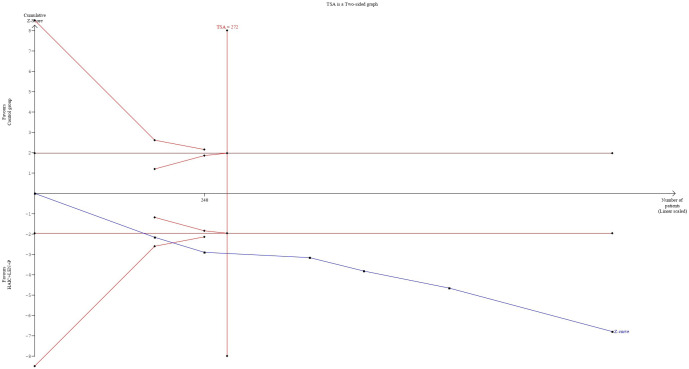
Trial sequential analysis result of objective response rate.

**Figure 9:**
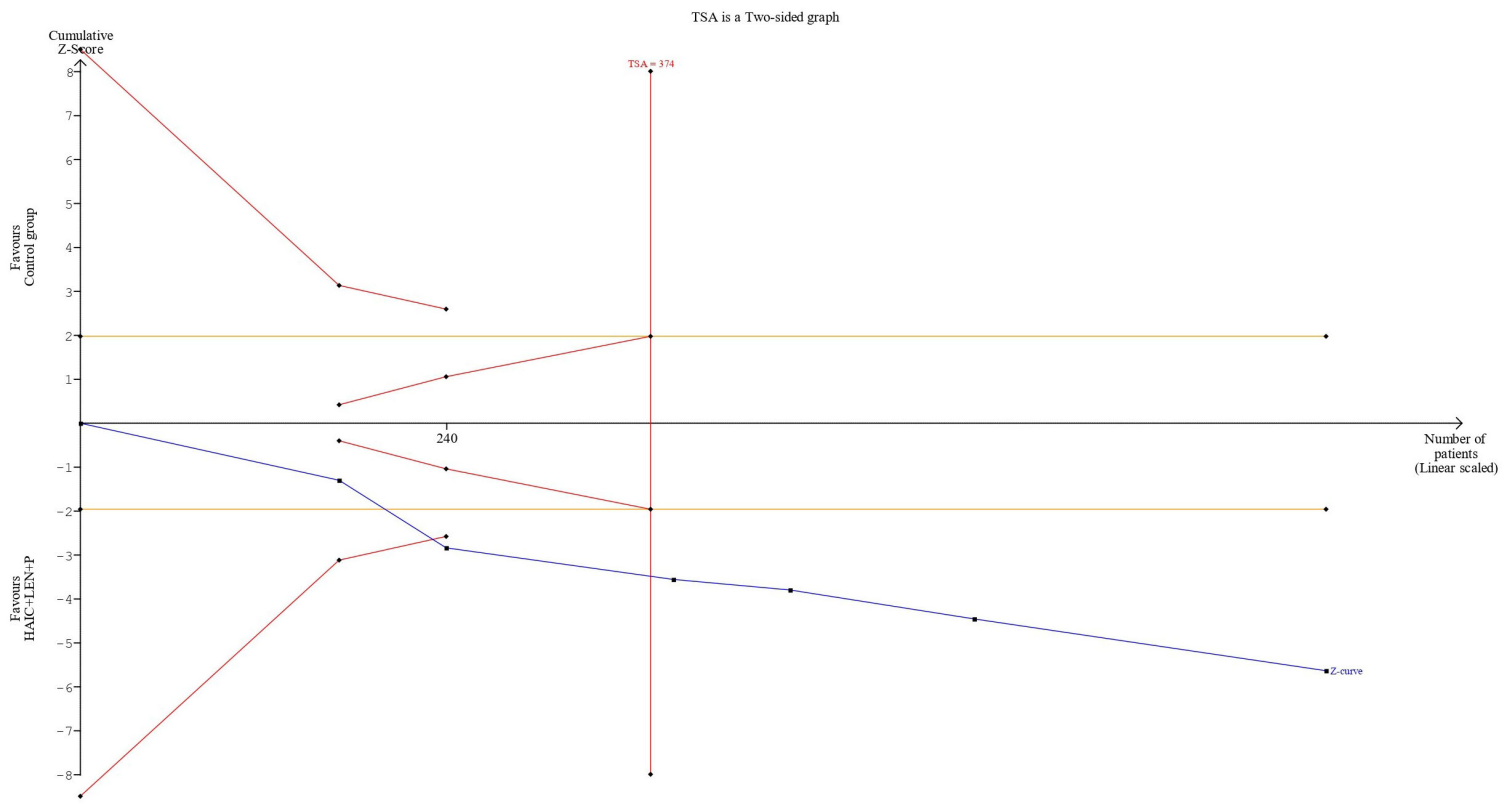
Trial sequential analysis result of disease control rate.

The cumulative sample size for ORR and DCR exceeded the optimal sample size for TSA analysis. In terms of the ORR, the cumulative Z-curve not only exceeded the conventional boundary but also surpassed the trial sequential monitoring boundary. This finding supports the evidence that combining HAIC with lenvatinib and PD-1 inhibitors resulted in an 82% increase in the ORR (**Figure 8**). For the DCR, the cumulative Z-curve also surpassed both the trial sequential monitoring boundary and the conventional thresholds. This finding indicates a 24% improvement in the DCR with the combined regimen compared to the control group. Moreover, this result suggests that additional trials may not be necessary to validate this benefit (**Figure 9**).

### 3.8 Publication bias

**Figure 10:**
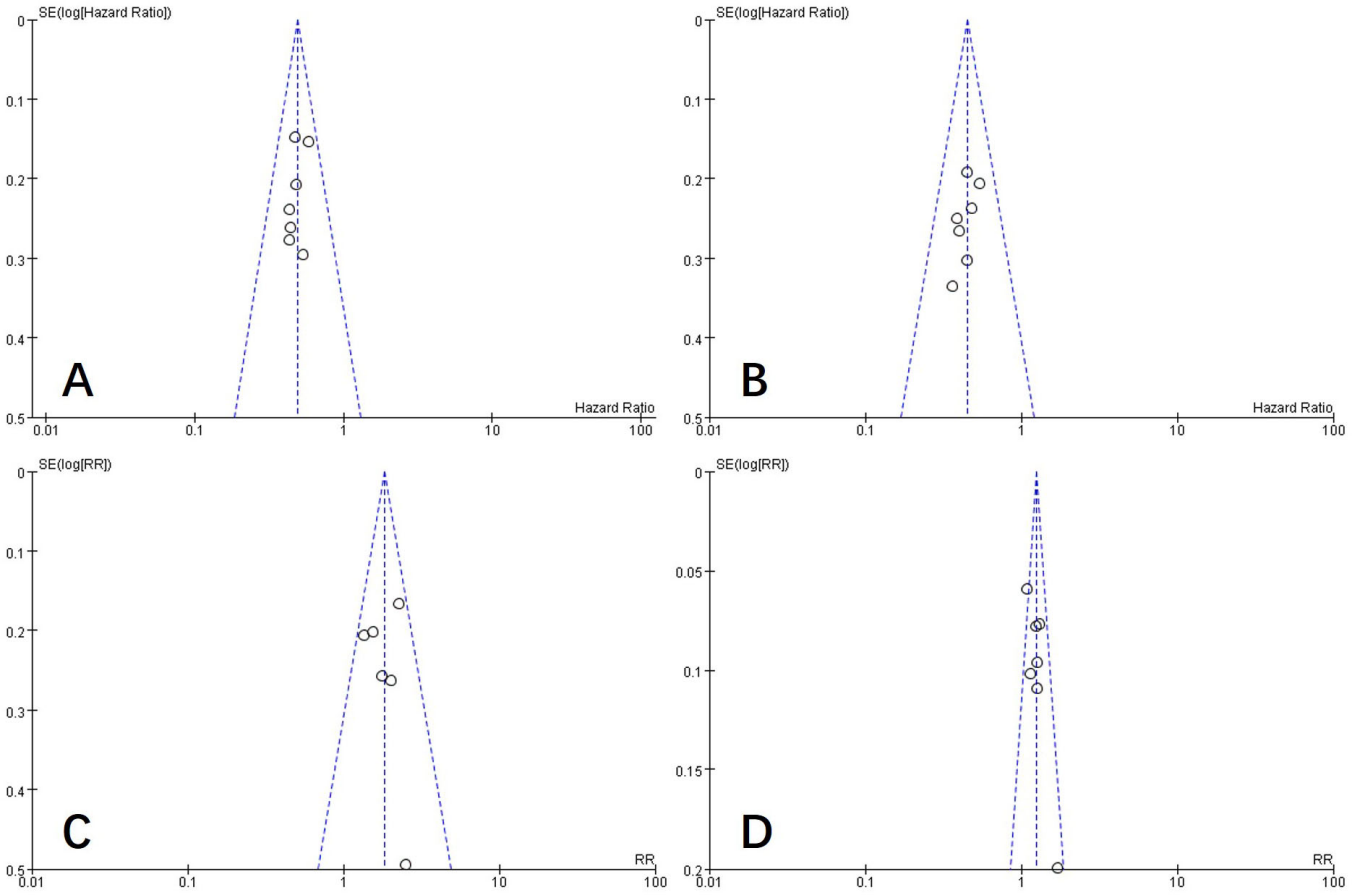
Funnel plot of PFS **(A)**, OS **(B)**, ORR **(C)**, and DCR **(D)**. PFS, progression-free survival; OS, overall survival; ORR, objective response rate; DCR, disease control rate.

To examine publication bias in this study, a funnel plot was constructed. As shown in **Figure 10**, the funnel plot appears symmetric, indicating an absence of publication bias in the literature included in this study.

## 4 Discussion

This meta-analysis investigated the efficacy of combining HAIC with lenvatinib and PD-1 inhibitors in the treatment of advanced HCC. Our outcomes revealed that this combination treatment notably increased OS, PFS, ORR, and DCR. TSA confirmed that the data available were adequate for making quantitative conclusions about the ORR and DCR.

For patients with early-stage HCC, surgical resection is regarded as the most effective treatment option in clinical practice. However, most patients are diagnosed at an advanced stage of the disease, missing the optimal treatment window. For patients with advanced, unresectable HCC, nonsurgical local or systemic treatments have become the primary options (27). According to the Barcelona Clinic Liver Cancer (BCLC) treatment strategy, hepatic arterial infusion chemotherapy (HAIC) is a commonly used treatment for advanced-stage liver cancer (4). Hsu et al. reported the efficacy of using a modified FOLFOX regimen as HAIC for refractory HCC patients who failed TACE treatment. The study revealed a median overall survival (OS) of 9 months and a median progression-free survival (PFS) of 3.7 months with this treatment (28). These results indicate that HAIC demonstrates favorable therapeutic efficacy even in advanced-stage hepatocellular carcinoma. In recent years, molecular targeted therapy has gradually become a hot topic in cancer research. Targeted drugs offer advantages such as broad antitumor effects, low systemic toxicity, and high specificity. These findings address the shortcomings of HAIC and provide additional treatment options for patients with advanced-stage liver cancer. Lenvatinib and PD-1 inhibitors are among these advancements, although Faivre et al. reported limited efficacy when these drugs are used individually (29). The latest guidelines from the American Society of Clinical Oncology (ASCO) on systemic treatment for HCC list tyrosine kinase inhibitors (TKIs) and immune checkpoint inhibitors (ICIs) as first-line treatment options. They also recommend that patients who receive ICIs as first-line treatment should consider TKIs for second-line therapy. The expert panel further suggests that ICIs are particularly beneficial for patients who are contraindicated or intolerant to TKIs (30). Additionally, Hu et al.’s study revealed poorer survival rates in patients with intrahepatic progression than in those with extrahepatic progression, underscoring the necessity for potent local treatments targeting intrahepatic lesions in second-line therapies (31). Our study underscores the possible synergistic benefits of the combination of these therapies. The observed enhancements in OS and PFS with the combination therapy indicate that it could represent a promising therapeutic option. This improvement has the potential to impact existing treatment standards.

Our study demonstrated that combined therapy with HAIC, lenvatinib, and PD-1 inhibitors can prolong overall survival (OS) and progression-free survival (PFS) in advanced HCC patients. This finding is consistent with findings from previous studies (32–34). This triple-therapy approach may prolong PFS and OS for several reasons. First, combination therapy has synergistic effects. HAIC delivers high concentrations of chemotherapy drugs directly into liver cancer lesions, effectively reducing the tumor burden (2). PD-1 inhibitors alleviate T-cell suppression, thereby enhancing immune responses to enable more effective recognition and attack of liver cancer cells (9). Previous studies have indicated that lenvatinib can normalize the tumor vasculature, reducing vascular permeability and tumor interstitial pressure. This normalization improves the delivery and distribution of chemotherapy drugs within tumors and potentially enhances the efficacy of local regional therapies (including TACE and HAIC) (35–37). Lenvatinib also increases PD-L1 expression in tumors, promoting immune cell infiltration into the tumor (19). Second, triple therapy helps overcome tumor resistance. Hepatocellular carcinoma often develops resistance to single treatments; the combination of HAIC with lenvatinib and PD-1 inhibitors can reduce tumor cell adaptability and resistance through different treatment mechanisms. Third, local and systemic control of tumors should be enhanced simultaneously. HAIC primarily targets localized liver tumors, effectively controlling the growth of primary lesions. Lenvatinib inhibits tumor angiogenesis and reduces the tumor blood supply, thereby suppressing tumor growth and distant metastasis. PD-1 inhibitors enhance immune responses systemically, combating tumor progression throughout the body.

In this study, we found that, compared with other treatments, combination therapy resulted in a higher overall response rate (ORR) and disease control rate (DCR). The TSA results for ORR and DCR provide sufficient evidence that combination therapy is beneficial. One of the core functions of TSA is to assess whether the existing studies provide sufficient evidence to support the current conclusions (38). In our analysis, the use of TSA indicates that the evidence for the effectiveness of combination therapy on ORR and DCR is already sufficiently robust, and further research is unlikely to significantly alter the current conclusions. This means that the existing sample size is adequate to draw conclusions regarding the benefits of combination therapy, and there is no need for additional studies to confirm these results. Furthermore, TSA determines whether statistical significance has been reached by setting critical boundaries, and in our study, the cumulative data surpassed these critical boundaries, further validating the advantages of combination therapy. In the study by He et al. (20), the ORR (67.6%) and DCR (90.1%) of HAIC combined with lenvatinib and PD-1 inhibitors were higher than the ORR (45.3%-65.33%) and DCR (72%-89%) reported in other studies. Additionally, 10 patients (14.1%) in the combination therapy group achieved complete remission. Notably, the subjects in He et al.’s study could be considered to have a poor prognosis, as the median size of the largest tumor was 10.9 cm and 74.5% of patients had portal vein tumor thrombus (PVTT). These findings suggest that patients with unresectable HCC have an increased chance of receiving radical treatment after receiving combination therapy, and even patients with a greater tumor burden can benefit from it.

For TACE-refractory HCC, previous studies have suggested that HAIC combined with sorafenib does not provide greater benefits than does sorafenib alone, indicating that HAIC may not be suitable for this population (39, 40). However, research by Lin et al. (21) and Diao et al. (22) on the efficacy of HAIC combined with lenvatinib and PD-1 inhibitors in these patients revealed that, compared with HAIC+lenvatinib or lenvatinib+PD-1 inhibitors, the combination treatment improved OS, PFS, ORR, and DCR without increasing the risk of complications. Therefore, the combination of HAIC, lenvatinib, and PD-1 inhibitors shows promise for improving survival in TACE-refractory patients and represents a treatment approach worth considering. Although these results are encouraging, further prospective, randomized controlled trials are needed to confirm these findings.

Our subgroup analysis revealed that, compared with various other treatment modalities, including HAIC combined with lenvatinib, lenvatinib plus PD-1 inhibitors, lenvatinib monotherapy, and HAIC combined with PD-1 inhibitors, HAIC combined with lenvatinib and PD-1 inhibitors demonstrated superior therapeutic efficacy. However, Chen et al. compared this combination therapy with TACE combined with HAIC, lenvatinib, and PD-1 inhibitors, and the results indicated that the quadruple therapy regimen significantly improved the survival of HCC patient (23). The possible reasons for these findings are that TACE therapy, in addition to providing local chemotherapy to target lesions, can also induce ischemic effects by blocking tumor blood vessels, leading to more pronounced tumor necrosis. A reduction in local tumor size may increase the efficacy of systemic treatments and prolong the duration of treatment (37). However, recent research by Hu et al. suggested that not all HCC patients benefit from additional local therapies (31). In frontline treatment, additional local therapies may not be suitable for rapidly progressing HCC. Controlled HCC posttreatment may indicate successful vascular normalization, which can further increase the efficacy of TACE or HAIC. Conversely, rapidly progressing HCC may suggest vascular normalization failure, resulting in the ineffectiveness of TACE or HAIC.

Recent guidelines have listed TKIs and ICIs as first-line treatment options, but evidence supporting the combination of multiple drugs remains insufficient. TKIs are only recommended as second-line treatment after ICIs fail as first-line therapy. The latest ASCO 2024 guidelines suggest that some new combination therapies may potentially improve disease control (30, 41). Both the 2023 American Association for the Study of Liver Diseases (AASLD) practice guidance on prevention, diagnosis, and treatment of hepatocellular carcinoma and the 2022 BCLC liver cancer treatment strategy emphasize the importance of combined and multidisciplinary approaches in the treatment of HCC (4, 42). Our research provides additional evidence supporting the efficacy of combining HAIC with lenvatinib and PD-1 inhibitors, particularly in patients with TACE-refractory disease, which could influence future treatment strategies. However, the patients included in our study were primarily from Asia, where the etiology of HCC differs from other regions, leading to variations in patient age distribution, tumor burden, staging, and other factors, which in turn may affect treatment outcomes. Therefore, our findings are more valuable for patients with similar clinical characteristics.

This study’s strengths lie in its rigorous methodology and the incorporation of a significant number of studies and participants, which enhance the reliability and robustness of our findings. Moreover, TSA adds further validation by mitigating the risk of false-positive results caused by random errors. Nonetheless, there are certain limitations. First, although the funnel plot indicates no significant publication bias in this study, and sensitivity and subgroup analyses have reduced the heterogeneity among the included studies, the potential heterogeneity in baseline characteristics of patients, disease staging, treatment regimens, and other factors could still influence the final results, given that all the included studies were retrospective. Moreover, due to limitations in the design of the studies themselves, biases in data selection and analysis could also affect the interpretation of the results. Therefore, future research should include more large-scale, well-designed randomized controlled trials to validate these findings. Additionally, studies that have not been published in peer-reviewed journals due to negative results or lack of efficacy conclusions (including gray literature such as clinical trial registries and conference abstracts) should be incorporated as much as possible to help minimize publication bias. Second, the studies we primarily included were all performed in Asia, which may affect the reliability and generalizability of the results, especially when considering differences in medical practices and patient characteristics between regions. To improve the external validity of the analysis, future studies should include research from different regions and backgrounds.

## 5 Conclusion

In conclusion, our study provides preliminary evidence for the combination of HAIC, lenvatinib, and PD-1 inhibitors in the treatment of advanced HCC, including in patients with heavy tumor burden and those who are refractory to TACE. This evidence may offer a new treatment approach to improve the prognosis of patients with advanced HCC. Larger-scale prospective and randomized controlled trials are needed in the future to validate and confirm these preliminary findings.

## Data Availability

The raw data supporting the conclusions of this article will be made available by the authors, without undue reservation.

## References

[1] Bray F Laversanne M Sung H Ferlay J Siegel RL Soerjomataram I . Global cancer statistics 2022: GLOBOCAN estimates of incidence and mortality worldwide for 36 cancers in 185 countries. CA: A Cancer J Clin. (2024) 74:229–63. doi: 10.3322/caac.21834 38572751

[2] Garg T Shrigiriwar A Habibollahi P Cristescu M Liddell RP Chapiro J . Intraarterial therapies for the management of hepatocellular carcinoma. Cancers. (2022) 14:3351. doi: 10.3390/cancers14143351 PMC9322128 35884412

[3] Llovet JM Montal R Sia D Finn RS . Molecular therapies and precision medicine for hepatocellular carcinoma. Nat Rev Clin Oncol. (2018) 15:599–616. doi: 10.1038/s41571-018-0073-4 30061739 PMC12452113

[4] Reig M Forner A Rimola J Ferrer-Fàbrega J Burrel M Garcia-Criado Á . BCLC strategy for prognosis prediction and treatment recommendation: The 2022 update. J Hepatol. (2022) 76:681–93. doi: 10.1016/j.jhep.2021.11.018 PMC8866082 34801630

[5] Al-Salama ZT Syed YY Scott LJ . Lenvatinib: A review in hepatocellular carcinoma. Drugs. (2019) 79:665–74. doi: 10.1007/s40265-019-01116-x 30993651

[6] Kudo M . Lenvatinib may drastically change the treatment landscape of hepatocellular carcinoma. Liver Cancer. (2018) 7:1–19. doi: 10.1159/000487148 29662829 PMC5892376

[7] He M Li Q Zou R Shen J Fang W Tan G . Sorafenib plus hepatic arterial infusion of oxaliplatin, fluorouracil, and leucovorin vs sorafenib alone for hepatocellular carcinoma with portal vein invasion. JAMA Oncol. (2019) 5:953–60. doi: 10.1001/jamaoncol.2019.0250 PMC6512278 31070690

[8] Long F Chen S Li R Lin Y Han J Guo J . Efficacy and safety of HAIC alone vs. HAIC combined with lenvatinib for treatment of advanced hepatocellular carcinoma. Med Oncol. (2023) 40:147. doi: 10.1007/s12032-023-02012-x 37043113

[9] Sangro B Sarobe P Hervás-Stubbs S Melero I . Advances in immunotherapy for hepatocellular carcinoma. Nat Rev Gastroenterol Hepatol. (2021) 18:525–43. doi: 10.1038/s41575-021-00438-0 PMC8042636 33850328

[10] Kim CG Kim C Yoon SE Kim KH Choi SJ Kang B . Hyperprogressive disease during PD-1 blockade in patients with advanced hepatocellular carcinoma. J Hepatol. (2021) 74:350–9. doi: 10.1016/j.jhep.2020.08.010 32810553

[11] Abou-Alfa GK Lau G Kudo M Chan SL Kelley RK Furuse J . Tremelimumab plus durvalumab in unresectable hepatocellular carcinoma. NEJM Evidence. (2022) 1:EVIDoa2100070. doi: 10.1056/EVIDoa2100070 38319892

[12] Ren Z Xu J Bai Y Xu A Cang S Du C . Sintilimab plus a bevacizumab biosimilar (IBI305) versus sorafenib in unresectable hepatocellular carcinoma (ORIENT-32): a randomised, open-label, phase 2-3 study. Lancet Oncol. (2021) 22:977–90. doi: 10.1016/S1470-2045(21)00252-7 34143971

[13] Llovet JM Lencioni R . mRECIST for HCC: Performance and novel refinements. J Hepatol. (2020) 72:288–306. doi: 10.1016/j.jhep.2019.09.026 31954493 PMC12452114

[14] Eisenhauer EA Therasse P Bogaerts J Schwartz LH Sargent D Ford R . New response evaluation criteria in solid tumours: revised RECIST guideline (version 1.1). Eur J Cancer. (2009) 45:228–47. doi: 10.1016/j.ejca.2008.10.026 19097774

[15] Lo CK-L Mertz D Loeb M . Newcastle-Ottawa Scale: comparing reviewers’ to authors’ assessments. BMC Med Res Methodol. (2014) 14:45. doi: 10.1186/1471-2288-14-45 PMC4021422 24690082

[16] Higgins JP Thompson SG Deeks JJ Altman DG . Measuring inconsistency in meta-analyses. BMJ. (2003) 327:557–60. doi: 10.1136/bmj.327.7414.557 PMC192859 12958120

[17] DerSimonian R Laird N . Meta-analysis in clinical trials revisited. Contemp Clin Trials. (2015) 45:139–45. doi: 10.1016/j.cct.2015.09.002 PMC4639420 26343745

[18] Mantel N Haenszel W . Statistical aspects of the analysis of data from retrospective studies of disease. J Natl Cancer Inst. (1959) 22:717–48. 13655060

[19] Mei J Tang YH Wei W Shi M Zheng L Li SH . Hepatic arterial infusion chemotherapy combined with PD-1 inhibitors plus lenvatinib versus PD-1 inhibitors plus lenvatinib for advanced hepatocellular carcinoma. Front Oncol. (2021) 11:618206. doi: 10.3389/fonc.2021.618206 33718175 PMC7947809

[20] He MK Liang RB Zhao Y Xu YJ Chen HW Zhou YM . Lenvatinib, toripalimab, plus hepatic arterial infusion chemotherapy versus lenvatinib alone for advanced hepatocellular carcinoma. Ther Adv Med Oncol. (2021) 13:17588359211002720. doi: 10.1177/17588359211002720 33854567 PMC8010824

[21] Lin LW Ke K Yan LY Chen R Huang JY . Efficacy and safety of hepatic artery infusion chemotherapy combined with tyrosine kinase inhibitors plus programmed death-1 inhibitors for hepatocellular carcinoma refractory to transarterial chemoembolization. Front Oncol. (2023) 13:1178428. doi: 10.3389/fonc.2023.1178428 37207144 PMC10189040

[22] Diao L Wang C You R Leng B Yu Z Xu Q . Hepatic arterial infusion chemotherapy combined with lenvatinib and PD-1 inhibitors versus lenvatinib and PD-1 inhibitors for HCC refractory to TACE. J Gastroenterol Hepatol. (2024) 39:746–53. doi: 10.1111/jgh.16463 38240156

[23] Chen S Shi F Wu Z Wang L Cai H Ma P . Hepatic arterial infusion chemotherapy plus lenvatinib and tislelizumab with or without transhepatic arterial embolization for unresectable hepatocellular carcinoma with portal vein tumor thrombus and high tumor burden: A multicenter retrospective study. J Hepatocell Carcinoma. (2023) 10:1209–22. doi: 10.2147/JHC.S417550 PMC10390715 37533600

[24] Chen S Xu B Wu Z Wang P Yu W Liu Z . Pembrolizumab plus lenvatinib with or without hepatic arterial infusion chemotherapy in selected populations of patients with treatment-naive unresectable hepatocellular carcinoma exhibiting PD-L1 staining: a multicenter retrospective study. BMC Cancer. (2021) 21:1126. doi: 10.1186/s12885-021-08858-6 34670506 PMC8527794

[25] Guan R Zhang N Deng M Lin Y Huang G Fu Y . Patients with hepatocellular carcinoma extrahepatic metastases can benefit from hepatic arterial infusion chemotherapy combined with lenvatinib plus programmed death-1 inhibitors. Int J Surg. (2024) 110:4062–73. doi: 10.1097/JS9.0000000000001378 PMC11254277 38549220

[26] Yu W Liu W Zhang K Chen S Wang X . Transarterial interventional therapy combined with tyrosine kinase inhibitors with or without anti-PD-1 antibodies as initial treatment for hepatocellular carcinoma with major portal vein tumor thrombosis: a single-center retrospective study. Cancer Immunol Immunother. (2023) 72:3609–19. doi: 10.1007/s00262-023-03511-6 PMC10991362 37566127

[27] Villarruel-Melquiades F Mendoza-Garrido ME García-Cuellar CM Sánchez-Pérez Y Pérez-Carreón JI Camacho J . Current and novel approaches in the pharmacological treatment of hepatocellular carcinoma. World J Gastroenterol. (2023) 29:2571–99. doi: 10.3748/wjg.v29.i17.2571 PMC10198058 37213397

[28] Hsu S-J Xu X Chen M-P Zhao Z-Y Wang Y Yin X . Hepatic arterial infusion chemotherapy with modified FOLFOX as an alternative treatment option in advanced hepatocellular carcinoma patients with failed or unsuitability for transarterial chemoembolization. Acad Radiol. (2021) 28:S157–S66. doi: 10.1016/j.acra.2021.01.024 33653656

[29] Faivre S Rimassa L Finn RS . Molecular therapies for HCC: Looking outside the box. J Hepatol. (2020) 72:342–52. doi: 10.1016/j.jhep.2019.09.010 31954496

[30] Gordan JD Kennedy EB Abou-Alfa GK Beal E Finn RS Gade TP . Systemic therapy for advanced hepatocellular carcinoma: ASCO guideline update. J Clin Oncol. (2024) 42:1830–50. doi: 10.1200/JCO.23.02745 38502889

[31] Hu Z Hu Z Zhan W Wu W Zhou Z Chen M . Efficacy of additional locoregional therapy based on systemic therapy after intrahepatic progression for BCLC stage B/C hepatocellular carcinoma: A real-world study. Int Immunopharmacol. (2024) 127:111413. doi: 10.1016/j.intimp.2023.111413 38118318

[32] Luo L Xiao Y Zhu G Huang A Song S Wang T . Hepatic arterial infusion chemotherapy combined with PD-1 inhibitors and tyrosine kinase inhibitors for unresectable hepatocellular carcinoma: A tertiary medical center experience. Front Oncol. (2022) 12. doi: 10.3389/fonc.2022.1004652 PMC9552711 36237309

[33] Xu Y Fu S Mao Y Huang S Li D Wu J . Efficacy and safety of hepatic arterial infusion chemotherapy combined with programmed cell death protein-1 antibody and lenvatinib for advanced hepatocellular carcinoma. Front Med. (2022) 9. doi: 10.3389/fmed.2022.919069 PMC9474651 36117969

[34] Chang X Wu H Ning S Li X Xie Y Shao W . Hepatic arterial infusion chemotherapy combined with lenvatinib plus humanized programmed death receptor-1 in patients with high-risk advanced hepatocellular carcinoma: A real-world study. J Hepatocellular Carcinoma. (2023) 10:1497–509. doi: 10.2147/JHC.S418387 PMC10493101 37701565

[35] Kano MR Komuta Y Iwata C Oka M Yt S Morishita Y . Comparison of the effects of the kinase inhibitors imatinib, sorafenib, and transforming growth factor-β receptor inhibitor on extravasation of nanoparticles from neovasculature. Cancer Science. (2009) 100:173–80. doi: 10.1111/j.1349-7006.2008.01003.x PMC11158202 19037999

[36] Jain RK . Normalization of tumor vasculature: an emerging concept in antiangiogenic therapy. Science. (2005) 307:58–62. doi: 10.1126/science.1104819 15637262

[37] Peng Z Fan W Zhu B Wang G Sun J Xiao C . Lenvatinib combined with transarterial chemoembolization as first-line treatment for advanced hepatocellular carcinoma: A phase III, randomized clinical trial (LAUNCH). J Clin Oncol. (2023) 41:117–27. doi: 10.1200/JCO.22.00392 35921605

[38] Wetterslev J Thorlund K Brok J Gluud C . Trial sequential analysis may establish when firm evidence is reached in cumulative meta-analysis. J Clin Epidemiol. (2008) 61:64–75. doi: 10.1016/j.jclinepi.2007.03.013 18083463

[39] Kudo M Ueshima K Yokosuka O Ogasawara S Obi S Izumi N . Sorafenib plus low-dose cisplatin and fluorouracil hepatic arterial infusion chemotherapy versus sorafenib alone in patients with advanced hepatocellular carcinoma (SILIUS): a randomised, open label, phase 3 trial. Lancet Gastroenterol Hepatol. (2018) 3:424–32. doi: 10.1016/S2468-1253(18)30078-5 29631810

[40] Vilgrain V Pereira H Assenat E Guiu B Ilonca AD Pageaux G-P . Efficacy and safety of selective internal radiotherapy with yttrium-90 resin microspheres compared with sorafenib in locally advanced and inoperable hepatocellular carcinoma (SARAH): an open-label randomised controlled phase 3 trial. Lancet Oncol. (2017) 18:1624–36. doi: 10.1016/S1470-2045(17)30683-6 29107679

[41] Lau G Obi S Zhou J Tateishi R Qin S Zhao H . APASL clinical practice guidelines on systemic therapy for hepatocellular carcinoma-2024. Hepatol Int. (2024) 18:1661–83. doi: 10.1007/s12072-024-10732-z 39570557

[42] Singal AG Llovet JM Yarchoan M Mehta N Heimbach JK Dawson LA . AASLD Practice Guidance on prevention, diagnosis, and treatment of hepatocellular carcinoma. Hepatology. (2023) 78:1922–65. doi: 10.1097/HEP.0000000000000466 PMC10663390 37199193

